# *N*-Heterocyclic carbene–palladium(II)-1-methylimidazole complex catalyzed Mizoroki–Heck reaction of aryl chlorides with styrenes

**DOI:** 10.3762/bjoc.8.222

**Published:** 2012-11-12

**Authors:** Ting-Ting Gao, Ai-Ping Jin, Li-Xiong Shao

**Affiliations:** 1 College of Chemistry and Materials Engineering, Wenzhou University, Chashan University Town, Zhejiang Province 325035, People’s Republic of China

**Keywords:** aryl chloride, Mizoroki–Heck reaction, *N*-heterocyclic carbene, palladium complex, synthetic method

## Abstract

A well-defined *N*-heterocyclic carbene–palladium(II)-1-methylimidazole [NHC-Pd(II)-Im] complex **1** was found to be an effective catalyst for the Mizoroki–Heck reaction of a variety of aryl chlorides with styrenes. Both activated and deactivated aryl chlorides work well to give the corresponding coupling products in good to excellent yields by using tetrabutylammonium bromide (TBAB) as the ionic liquid.

## Introduction

The palladium-catalyzed reaction between organic halides and alkenes, the Mizoroki–Heck reaction, is one of the most versatile methods for the formation of carbon–carbon bonds [1–7]. Usually, in order to achieve the highest efficiency of the palladium-catalyzed Mizoroki–Heck reaction, toxic, air-sensitive and expensive phosphine ligands are introduced to facilitate the corresponding transformations [8–9]. In order to overcome the drawbacks associated with the phosphine ligands, the development of alternative stable, inexpensive and easily available phosphine-free ligands is still in great demand.

In the meantime, *N*-heterocyclic carbenes (NHCs), possessing the advantages of being stronger σ-donors and weaker π-acceptors than traditional phosphine ligands, which can then increase the catalytic activity of the metal centre and the stability of the NHC–metal complexes, have attracted growing attention in the metal-catalyzed carbon–carbon and carbon–heteroatom bond-formation reactions during the past two decades [10–15]. Consequently, since the seminal papers reported by Herrmann and co-workers [16–17], the NHC–Pd complexes have become a strong challenge to the phosphine–metal complexes in the Mizoroki–Heck reaction. Among the electrophiles participating in the NHC–Pd-complex-catalyzed Mizoroki–Heck reaction, organic iodides and bromides, which can be easily activated by the metal centre, are usually used as good partners [18–25]. In contrast to the common electrophiles such as organic iodides and bromides, organic chlorides are the most attractive in the Mizoroki–Heck reaction toward industrial applications due to their lower cost and the widest availability [26–28]. However, organic chlorides, especially the deactivated ones, are generally unreactive in the NHC–Pd-complex-catalyzed Mizoroki-Heck reaction [29–31]. Furthermore, synthetic routes for the above mentioned NHC–Pd complexes that showed good catalytic activity toward activated aryl chlorides, are all lengthy [32–36]. Therefore, although the progress has been achieved for the NHC–Pd-complex-catalyzed Mizoroki–Heck reaction of aryl chlorides, the drive to develop easily available and widely applicable complexes with broad substrate scope, is still a current topic of interest.

**Figure 1:**
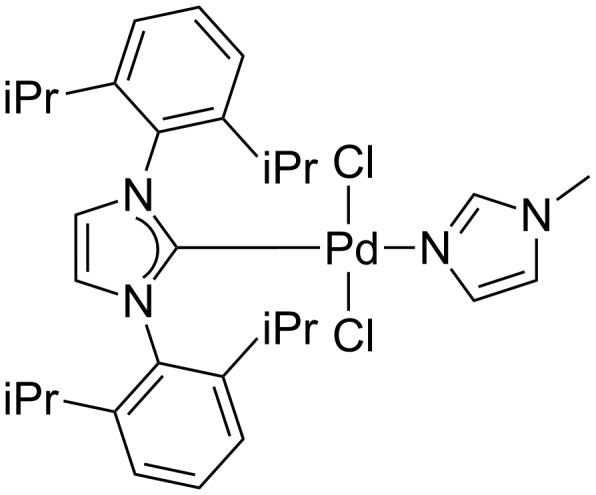
NHC-Pd(II)-Im complex **1**.

Recently, we have demonstrated that the well-defined *N*-heterocyclic carbene–palladium(II)-1-methylimidazole [NHC-Pd(II)-Im] complex **1** (Figure 1), which can be easily prepared from commercially available PdCl_2_, IPr**^.^**HCl [1,3-bis(2,6-diisopropylphenyl)imidazolium chloride], and 1-methylimidazole in a one-step procedure in high yield, was an effective catalyst in C–C and C–N bond-formation reactions [37–44]. In our continuing investigations on the further applications of this complex in organic synthesis, we found that complex **1** was an active catalyst for the Mizoroki–Heck reaction of aryl chlorides, including both activated and deactivated aryl chlorides, with styrenes performed under air. Herein, we wish to report these results in detail.

## Results and Discussion

Initially, the model reaction between chlorobenzene (**2a**) (0.75 mmol) and styrene (**3a**) (0.90 mmol) was carried out in common polar solvents, such as *N*,*N*-dimethylformamide, *N*,*N*-dimethylacetamide and dimethylsulfoxide, in the presence of different inorganic bases and NHC-Pd(II)-Im complex **1** (1.0 mol %) at 150 °C for 24 h. To our disappointment, almost no desired product **4a** was observed in all cases. It was reported that tetrabutylammonium bromide (TBAB), as a simple ionic liquid, possessing the advantages of low price and easy availability, can facilitate the NHC–Pd-complex-catalyzed Mizoroki–Heck reaction [29–31,33]. Thus, we then turned our interest to carrying out the reaction between chlorobenzene (**2a**) and styrene (**3a**) in the presence of NHC-Pd(II)-Im **1** using TBAB as the solvent at 140 °C (Table 1). As can be seen from Table 1, all reactions took place smoothly under air to give the desired coupling product **4a** in low to excellent yields within 12 h in the presence of any of the bases tested. To our pleasure, the best result can be achieved when Cs_2_CO_3_ was used as the base (Table 1, entry 1) [45].

**Table 1:** Optimization for the NHC-Pd(II)-Im complex **1** catalyzed reaction of chlorobenzene (**2a**) with styrene (**3a**).

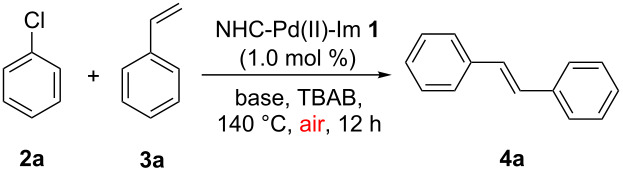		

Entry^a^	Base	Yield (%)^b^

1	Cs_2_CO_3_	94
2	NaOH	54
3	KOH	67
4	NaHCO_3_	80
5	KHCO_3_	22
6	Na_2_CO_3_	52
7	K_2_CO_3_	34
8	KO*t*-Bu	15
9	NaO*t*-Bu	44
10	LiO*t*-Bu	85

^a^All reactions were carried out by using **2a** (0.75 mmol), **3a** (0.9 mmol), base (2.0 equiv), **1** (1.0 mol %), TBAB (2.0 g) at 140 °C for 12 h. ^b^Isolated yields.

The optimal reaction conditions (Table 1, entry 1) were then applied to a variety of aryl chlorides and styrenes to investigate the generality (Table 2). As can be seen from Table 2, most reactions proceeded well to give products **4** in good to high yields. For instance, in the first round, the reactions between various aryl chlorides **2** and styrene (**3a**) were examined (Table 2, entries 1–7). It seems that all of the activated and deactivated aryl chlorides work well under the optimal conditions to give the products **4b**, **4c**, **4e**, **4f** and **4g** in good to high yields (Table 2, entries 1, 2, 4, 5 and 6). 2-Methylphenyl chloride (**2d**) gave product **4d** in a slightly lower yield (71%) probably due to its steric hindrance (Table 2, entry 3). The reaction between 2-chlorothiophene (**2h**) and styrene (**3a**) also gave product **4h** in 63% yield (Table 2, entry 7). In a second round, the reactions between chlorobenzene (**2a**) and various styrenes **3** were also investigated (Table 2, entries 8–13). It also seems that the electronic effect of the substituents on the styrenes did not affect the reactions, and all reactions took place smoothly to give products **4** in good yields (Table 2, entries 8–12). The reaction between chlorobenzene (**2a**) and 2,4-dimethylstyrene (**3g**) only gave product **4l** in 67% yield, which may also be due to the steric effect. In addition, acceptable yields can be achieved from the reactions of 2-chlorothiophene (**2h**) with 4-fluorostyrene (**3b**) and 4-methylstyrene (**3c**), respectively (Table 2, entries 19 and 20).

**Table 2:** NHC-Pd(II)-Im complex **1** catalyzed Mizoroki–Heck reactions of aryl chlorides **2** with styrenes **3**.

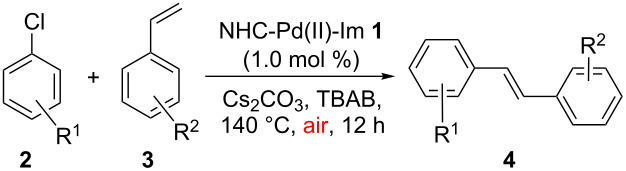			

Entry^a^	**2** (R^1^)	**3** (R^2^)	Yield (%)^b^

1	**2b** (3-MeO)	**3a** (H)	**4b**, 85
2	**2c** (3-Me)	**3a**	**4c**, 94
3	**2d** (2-Me)	**3a**	**4d**, 71
4	**2e** (4-F)	**3a**	**4e**, 91
5	**2f** (4-Me)	**3a**	**4f**, 80
6	**2g** (4-NO_2_)	**3a**	**4g**, 89
7^c^	**2h**	**3a**	**4h**, 63
8	**2a** (H)	**3b** (4-F)	**4e**, 81
9	**2a**	**3c** (4-Me)	**4f**, 85
10	**2a**	**3d** (4-MeO)	**4i**, 83
11	**2a**	**3e** (3-F)	**4j**, 81
12	**2a**	**3f** (4-*t*-Bu)	**4k**, 80
13	**2a**	**3g** (2,4-Me_2_)	**4l**, 67
14	**2c** (3-Me)	**3c**	**4m**, 85
15	**2g**	**3b**	**4n**, 90
16	**2g**	**3c**	**4o**, 89
17	**2g**	**3e**	**4p**, 95
18	**2g**	**3f**	**4q**, 86
19^c^	**2h**	**3b**	**4r**, 53
20^c^	**2h**	**3c**	**4s**, 51

^a^All reactions were carried out by using **2** (0.75 mmol), **3** (0.9 mmol), Cs_2_CO_3_ (2.0 equiv), **1** (1.0 mol %), TBAB (2.0 g) at 140 °C for 12 h. ^b^Isolated yields. ^c^
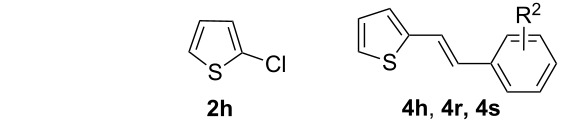

## Conclusion

In summary, an easily available NHC-Pd(II)-Im complex **1** showed efficient catalytic activity toward the Mizoroki–Heck reaction between aryl chlorides and styrenes under air at 140 °C within 12 h. Various activated and deactivated aryl chlorides work well under the optimal reaction conditions. In addition, TBAB, as a “green solvent” and one of the most advantageous ionic liquids due to its low price and availability, was used as the solvent to facilitate the reaction [46–48]. Compared to the previously reported NHC–Pd(II) complexes used in the Mizoroki–Heck reaction with aryl chlorides as the substrates, the method reported in this paper possesses the advantages of easy availability of the catalyst and broader substrate applicability.

## Supporting Information

File 1 General procedure for the NHC-Pd(II)-Im complex **1** catalyzed Mizoroki–Heck reaction, characterization data and copies of spectra.
