# Supplementary material for: N-Heterocyclic carbene–palladium(II)-1-methylimidazole complex catalyzed Mizoroki–Heck reaction of aryl chlorides with styrenes
Source: Beilstein J Org Chem. 2012 Nov 12;8:1916–9. doi: 10.3762/bjoc.8.222 (PMC3511031; doi:10.3762/bjoc.8.222)
Supplement: File 1 — General procedure for the NHC-Pd(II)-Im complex 1 catalyzed Mizoroki–Heck reaction, characterization data and copies of spectra. [file Beilstein_J_Org_Chem-08-1916-s001.pdf]

Supporting Information
for
N-Heterocyclic carbene–palladium(II)-1-methylimidazole complex catalyzed Mizoroki–Heck reaction of aryl chlorides with styrenes

Ting-Ting Gao, Ai-Ping Jin and Li-Xiong Shao*

Address: College of Chemistry and Materials Engineering, Wenzhou University, Chashan University Town, Zhejiang Province 325035, People's Republic of China.

Email: Li-Xiong Shao* - Shaolix@wzu.edu.cn

* Corresponding author

General procedure for the NHC-Pd(II)-Im complex 1 catalyzed Mizoroki–Heck reaction, characterization data and copies of spectra

Table of contents

General remarks S2

General procedure S2

Characterization data and spectra S3

References S22

General remarks**:** ^{1}H and ^{13}C NMR spectra were recorded on a Bruker Avance-300 MHz or Bruker Avance-500 MHz spectrometer for solutions in $CDCl_3$ with tetramethylsilane (TMS) as an internal standard; *J*-values are in Hz. Commercially obtained reagents were used without further purification. Flash column chromatography was carried out using Huanghai 300–400 mesh silica gel at increased pressure.

General procedure**:** Under air, TBAB (2.0 g), Cs_2CO_3 (488.7 mg, 2.0 equiv), NHC-Pd(II)-Im complex **1** (4.9 mg, 1.0 mol %), styrenes **3** (0.90 mmol) and aryl chlorides **2** (0.75 mmol) were successively added into a sealed tube. Then the mixture was stirred at 140 °C for 12 h. After cooling to room temperature, the reaction mixture was diluted with ethyl acetate, washed with 1 M HCl (15 mL x 2), brine (15 mL x 2) and dried over anhydrous Na_2SO_4. The solvent was evaporated in vacuo and then purified by flash column chromatography on silica gel to give the pure products.

Characterization data and spectra

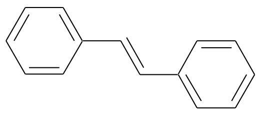

Compound **4a** [1]: a white solid; ^{1}H NMR (300 MHz, $CDCl_3$) δ 7.08 (s, 2H), 7.20–7.35 (m, 6H), 7.47–7.50 (m, 4H); ^{13}C NMR (125 MHz, $CDCl_3$) δ 126.5, 127.6, 128.66, 128.70, 137.3.

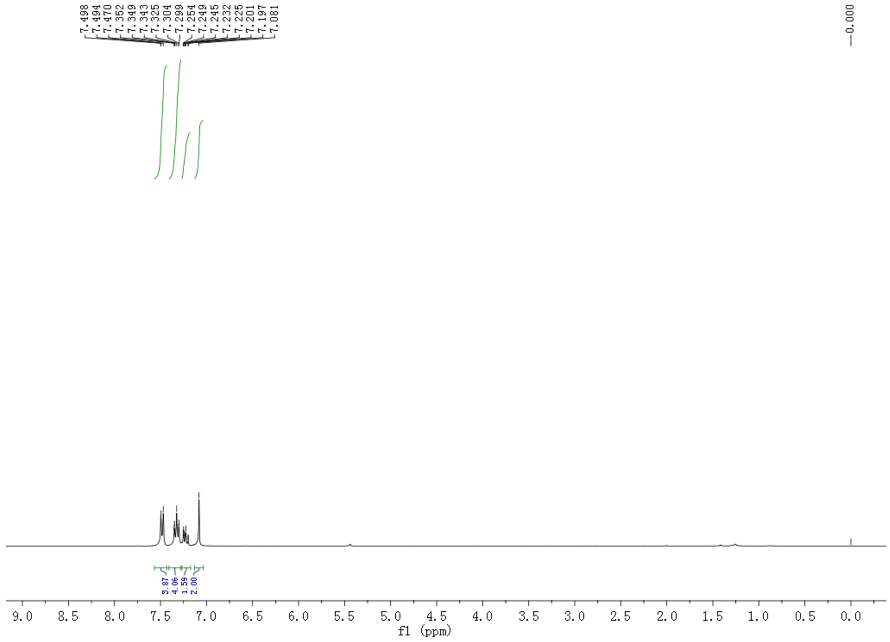

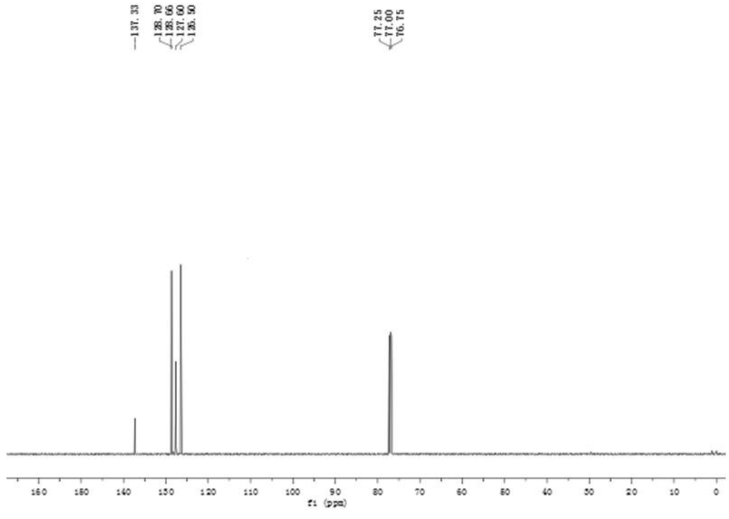

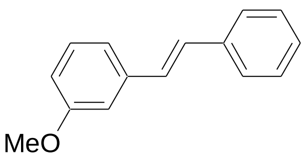

Compound **4b** [1]: a colorless liquid; ^{1}H NMR (500 MHz; $CDCl_3$) δ 3.82 (s, 3H), 6.81 (d, J = 8.0 Hz, 1H), 7.04–7.11 (m, 4H), 7.19–7.35 (m, 4H), 7.49 (d, J = 8.0 Hz, 2H); ^{13}C NMR (125 MHz, $CDCl_3$) δ 55.3, 111.8, 113.3, 119.3, 126.5, 127.7, 128.6, 128.7, 129.6, 137.3, 138.8, 159.9.

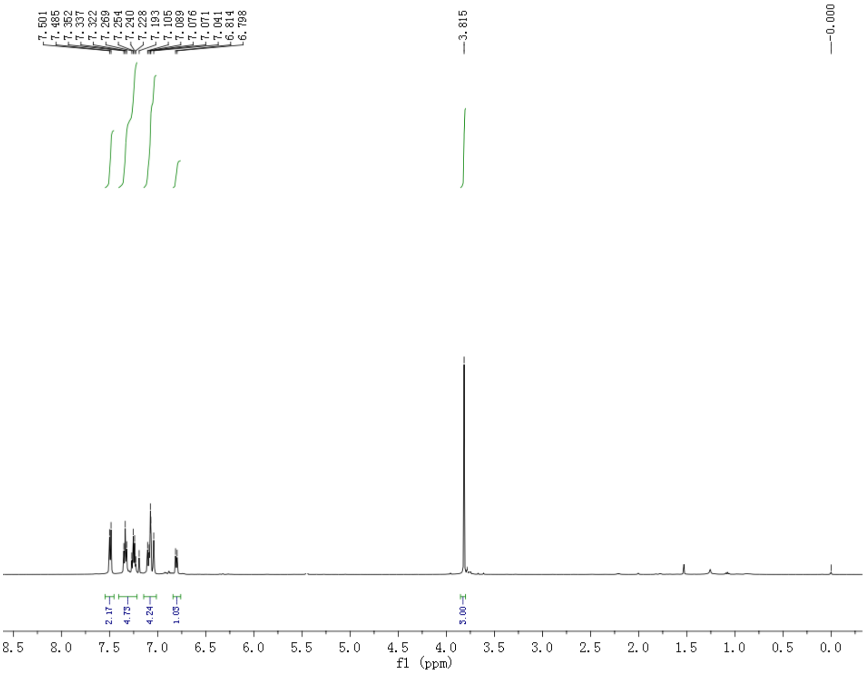

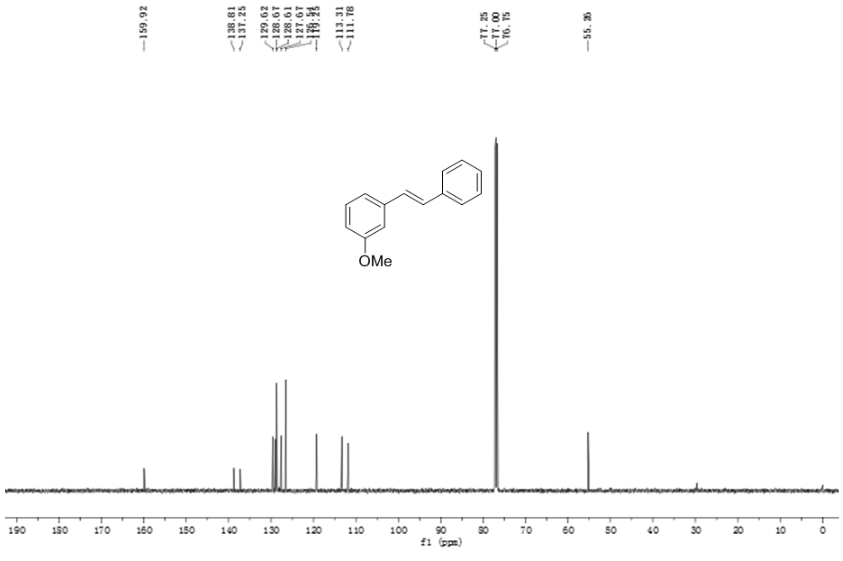

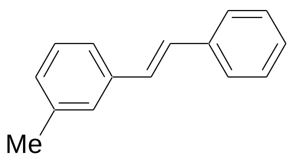

Compound **4c** [2]: a white solid; ^{1}H NMR (300 MHz, $CDCl_3$) δ 2.34 (s, 3H), 7.00–7.11 (m, 3H), 7.18–7.34 (m, 6H), 7.46–7.49 (m, 2H); ^{13}C NMR (125 MHz, $CDCl_3$) δ 21.4, 123.7, 126.4, 127.2, 127.5, 128.40, 128.44, 128.5, 128.6, 128.8, 137.2, 137.4, 138.1.

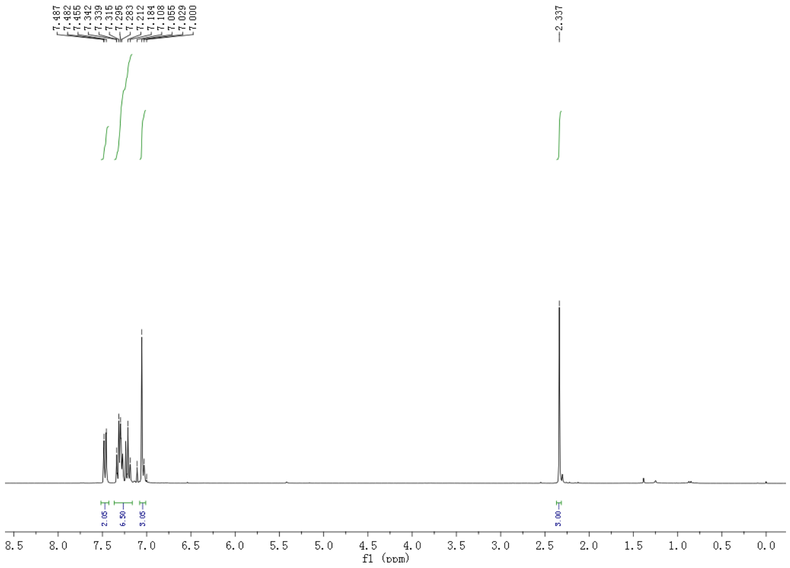

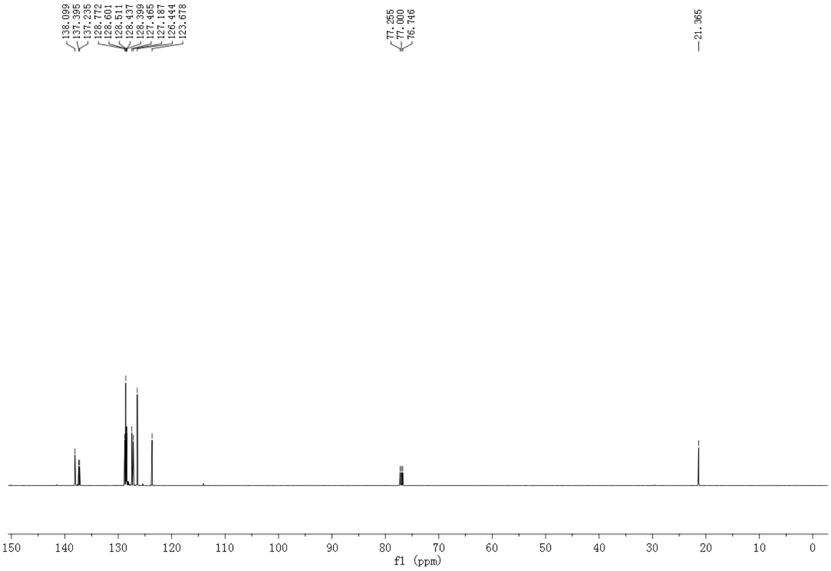

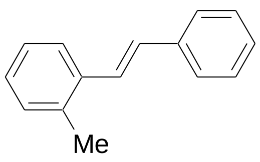

Compound **4d** [2]: a colorless liquid; ^{1}H NMR (300 MHz, $CDCl_3$) δ 2.38 (s, 3H), 6.96 (d, J = 15.9 Hz, 1H), 7.13–7.34 (m, 7H), 7.47–7.57 (m, 3H); ^{13}C NMR (125 MHz, $CDCl_3$) δ 19.9, 125.4, 126.2, 126.55, 126.57, 127.5, 127.6, 128.7, 130.0, 130.4, 135.8, 136.4, 137.7.

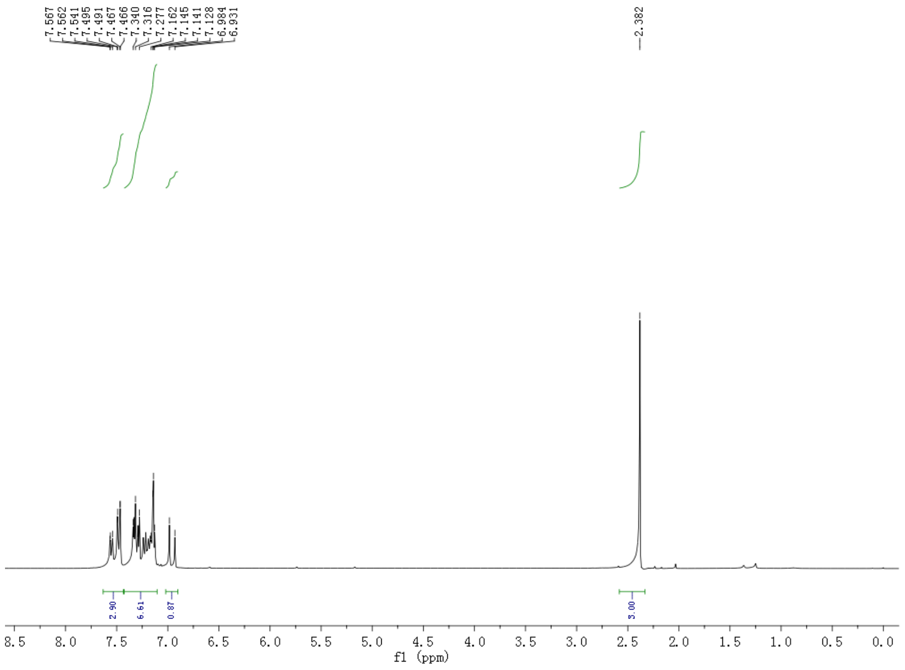

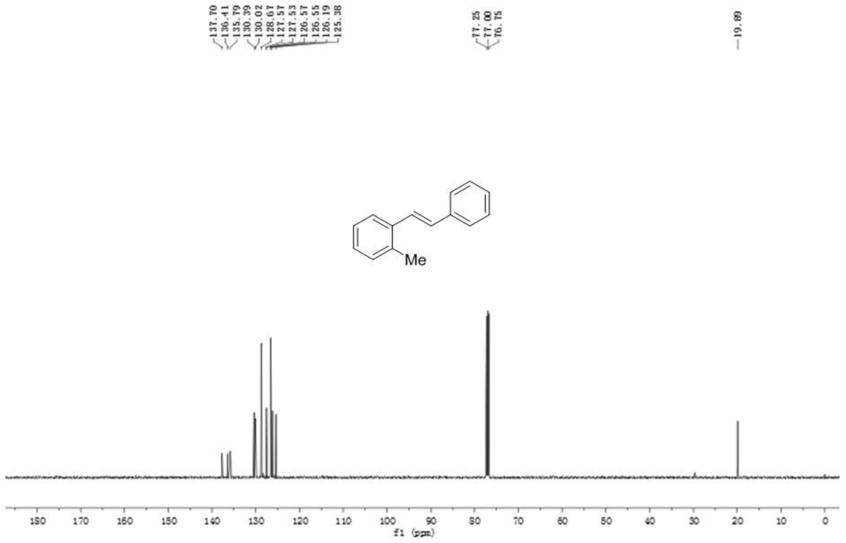

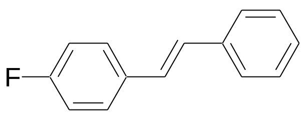

Compound **4e** [3]: ^{1}H NMR (300 MHz, $CDCl_3$) δ 6.97–7.10 (m, 4H), 7.23–7.44 (m, 3H), 7.48–7.51 (m, 4H); ^{13}C NMR (75 MHz, $CDCl_3$) δ 115.5 (d, $J_{C\text{-}F}$ = 21.6 Hz), 126.4, 127.4, 127.6, 127.9 (d, $J_{C\text{-}F}$ = 8.0 Hz), 128.4, 128.7, 133.4, 137.1, 162.2 (d, $J_{C\text{-}F}$ = 245.6 Hz).

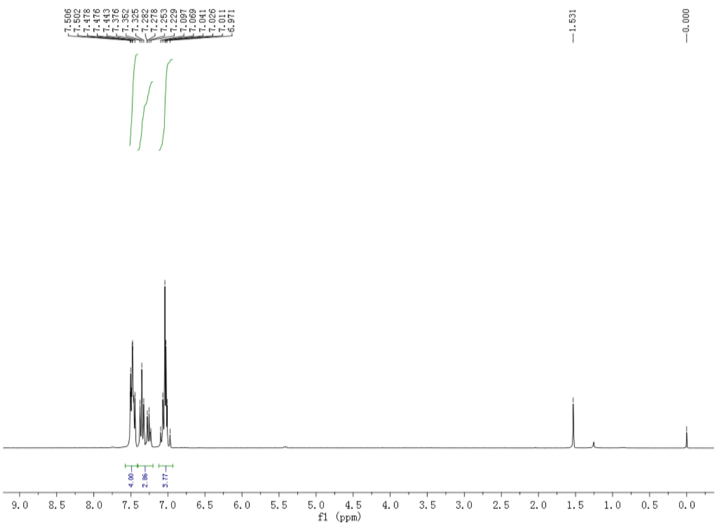

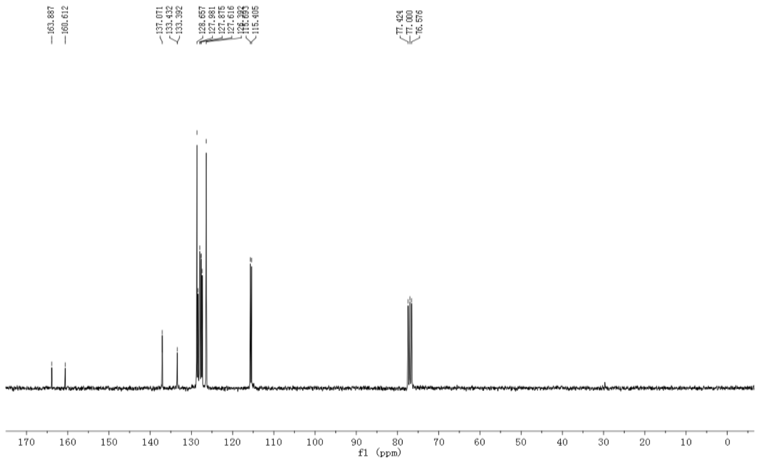

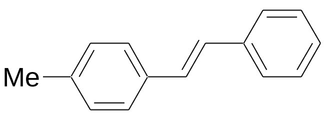

Compound **4f** [4]: a white solid; ^{1}H NMR (300 MHz, $CDCl_3$) δ 2.35 (s, 3H), 7.04 (d, *J* = 16.5 Hz, 1H), 7.10 (d, *J* = 16.5 Hz, 1H), 7.16 (d, *J* = 7.8 Hz, 2H), 7.23 (t, *J* = 7.8 Hz, 1H), 7.34 (t, *J* = 7.8 Hz, 2H), 7.41 (d, *J* = 7.8 Hz, 2H), 7.49 (d, *J* = 7.5 Hz, 2H); ^{13}C NMR (125 MHz, $CDCl_3$) δ 21.2, 126.37, 126.41, 127.4, 127.7, 128.61, 128.62, 129.4, 134.5, 137.48, 137.50.

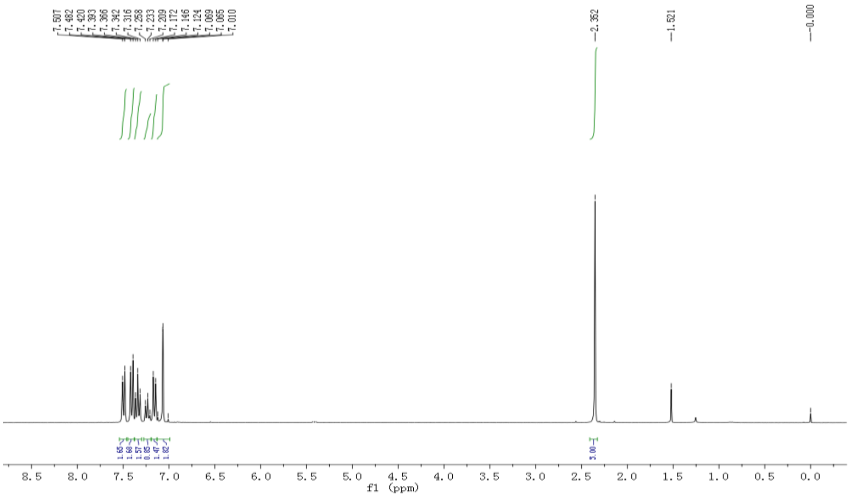

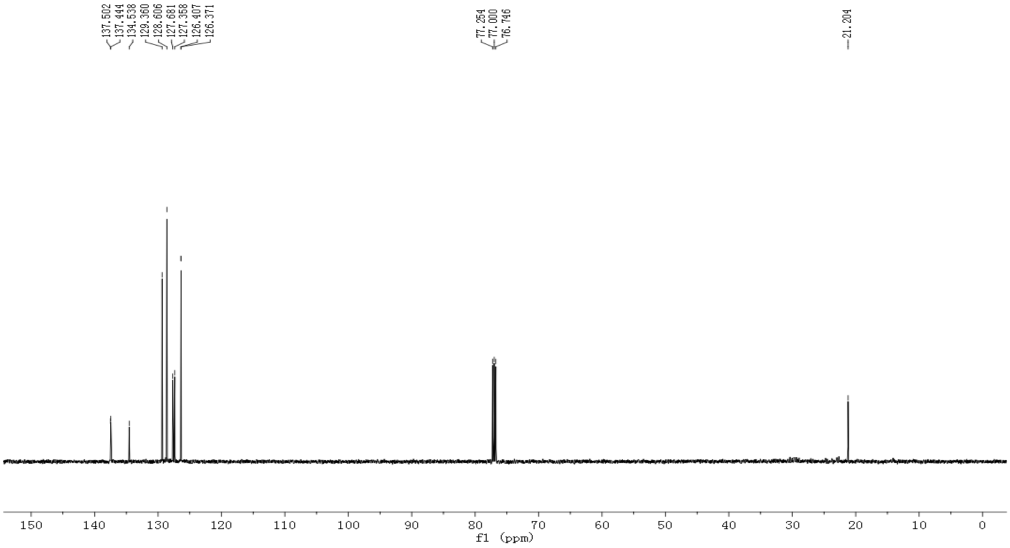

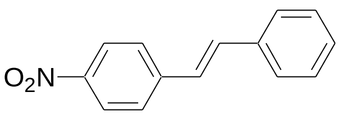

Compound **4g** [2]: a yellow solid; ^{1}H NMR (300 MHz, $CDCl_3$) δ 7.14 (d, *J* = 16.5 Hz, 1H), 7.28 (d, *J* = 16.5 Hz, 1H), 7.34–7.43 (m, 3H), 7.56 (d, *J* = 7.5 Hz, 2H), 7.63 (d, *J* = 8.4 Hz, 2H), 8.22 (d, *J* = 8.4 Hz, 2H); ^{13}C NMR (75 MHz, $CDCl_3$) δ 124.1, 126.2, 126.8, 127.0, 128.8, 128.9, 133.3, 136.1, 143.8, 146.7.

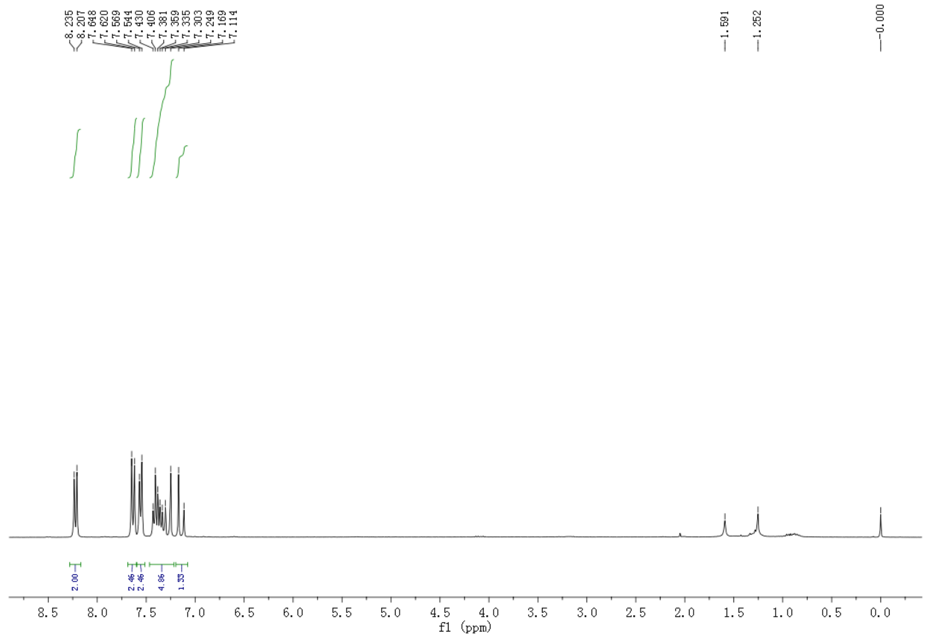

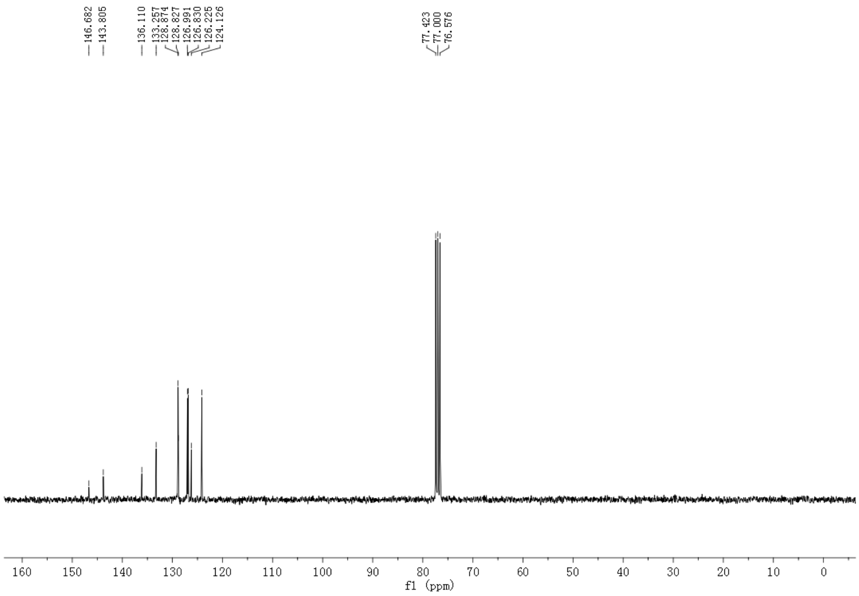

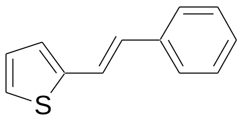

Compound **4h** [5]: a yellow solid; ^{1}H NMR (300 MHz, $CDCl_3$) δ 6.93 (d, *J* = 15.9 Hz, 1H), 7.00 (dd, *J* = 3.6, 5.1 Hz, 1H), 7.06–7.07 (m, 1H), 7.18–7.37 (m, 5H), 7.45–7.48 (m, 2H); ^{13}C NMR ($CDCl_3$, 125 MHz) δ 121.8, 124.3, 126.1, 126.3, 127.6, 128.3, 128.7, 136.9, 142.9.

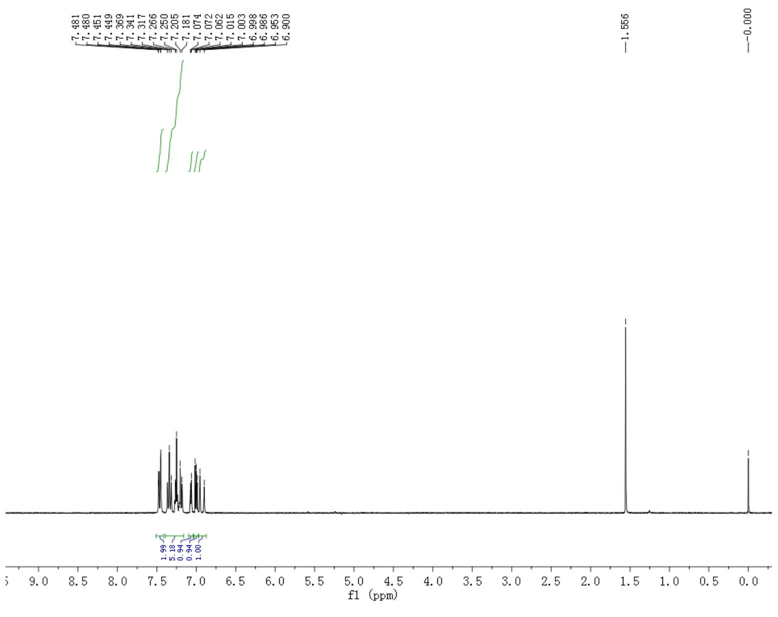

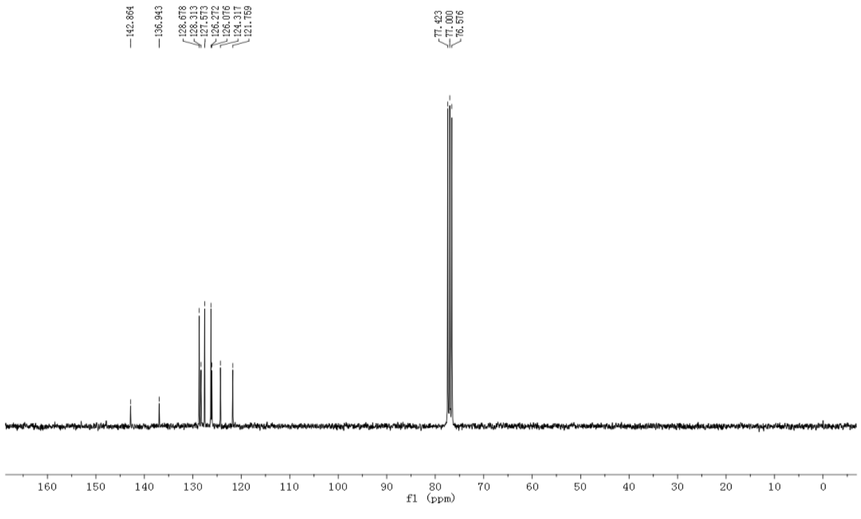

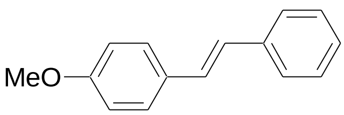

Compound **4i** [5]: a white solid; ^{1}H NMR (500 MHz, $CDCl_3$) δ 3.83 (s, 3H), 6.90 (d, *J* = 8.5 Hz, 2H), 6.97 (d, *J* = 16.0 Hz, 1H), 7.07 (d, *J* = 16.0 Hz, 1H), 7.23 (t, *J* = 7.5 Hz, 1H), 7.34 (t, *J* = 7.5 Hz, 2H), 7.45 (d, *J* = 8.5 Hz, 2H), 7.49 (d, *J* = 8.5 Hz, 2H); ^{13}C NMR (125 MHz, $CDCl_3$) δ 55.3, 114.1, 126.2, 126.6, 127.2, 127.7, 128.2, 128.6, 130.2, 137.7, 159.3.

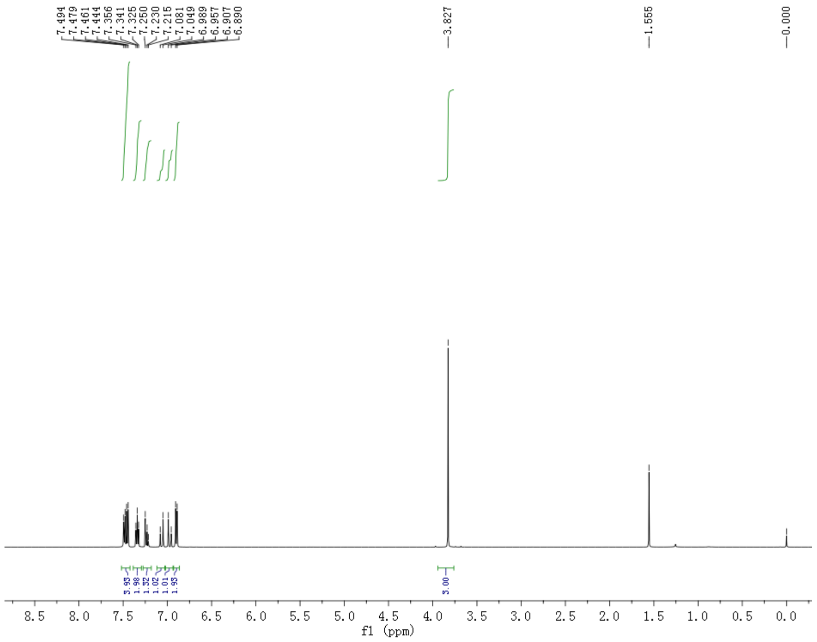

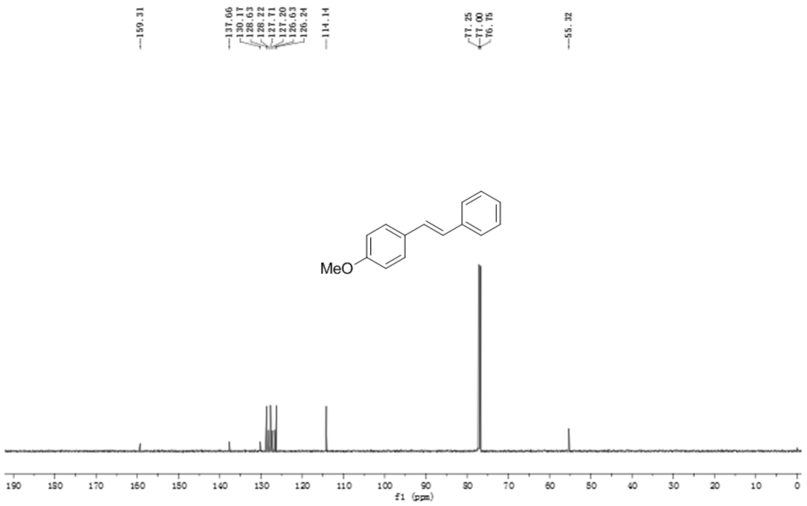

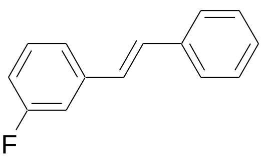

Compound **4j** [6]: a white solid; ^{1}H NMR (500 MHz, $CDCl_3$) δ 6.92 (t, J = 8.0 Hz, 1H), 7.01 (d, J = 15.6 Hz, 1H), 7.07 (d, J = 15.6 Hz, 1H), 7.17–7.35 (m, 6H), 7.47 (d, J = 7.5 Hz, 2H); ^{13}C NMR (125 MHz, $CDCl_3$) δ 112.7 (d, $J_{C\text{-}F}$ = 21.8 Hz), 114.3 (d, $J_{C\text{-}F}$ = 21.4 Hz), 122.4, 126.6, 127.4, 127.9, 128.7, 130.0, 136.8, 139.7 (d, $J_{C\text{-}F}$ = 7.7 Hz), 163.1 (d, $J_{C\text{-}F}$ = 243.8 Hz).

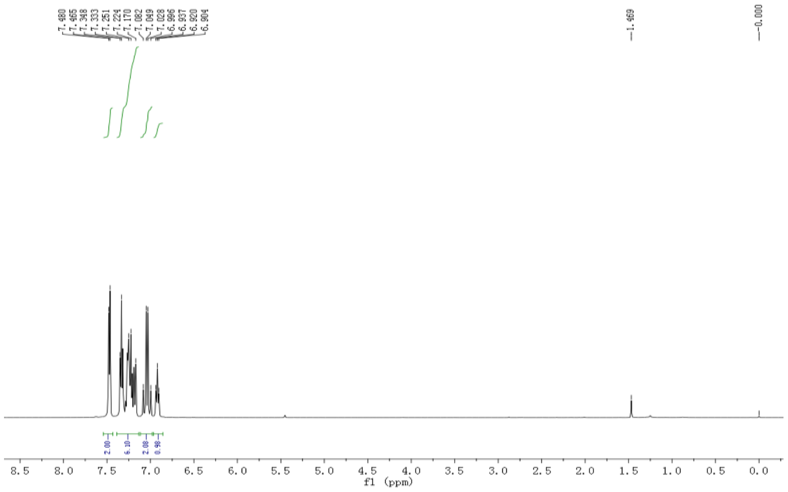

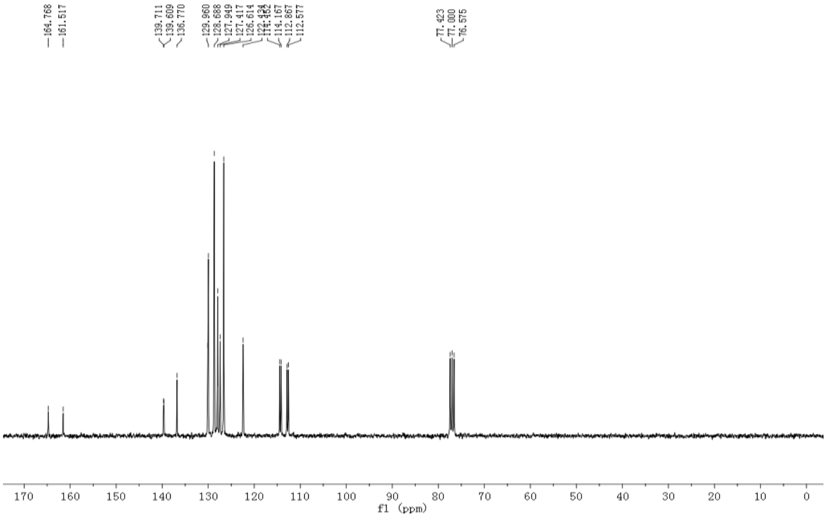

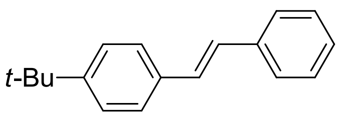

Compound **4k** [7]: a white solid; ^{1}H NMR (300 MHz, $CDCl_3$) δ 1.32 (s, 9H), 7.05 (d, *J* = 16.5 Hz, 1H), 7.09 (d, *J* = 16.5 Hz, 1H), 7.22 (t, *J* = 7.0 Hz, 1H), 7.31–7.37 (m, 4H), 7.44 (d, *J* = 8.0 Hz, 2H), 7.48 (d, *J* = 8.0 Hz, 2H); ^{13}C NMR (125 MHz, $CDCl_3$) δ 31.3, 34.6, 125.6, 126.2, 126.4, 127.4, 127.9, 128.5, 128.6, 134.5, 137.5, 150.7.

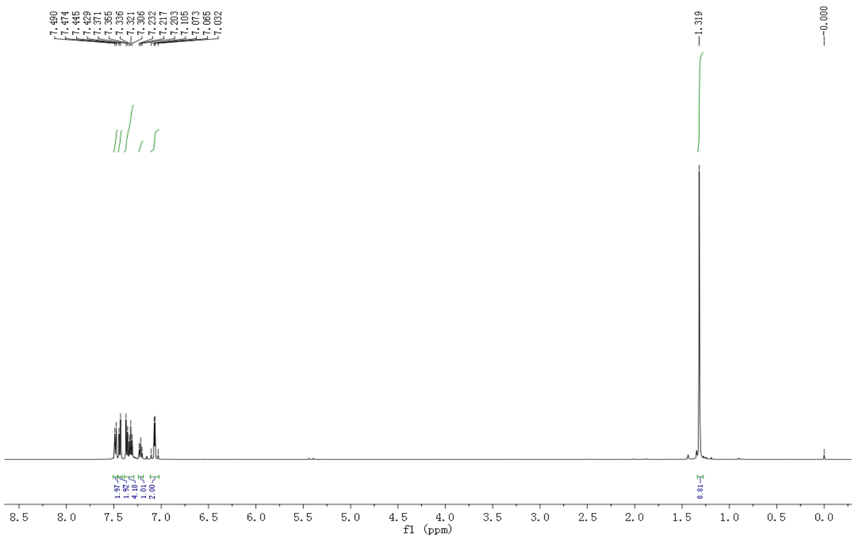

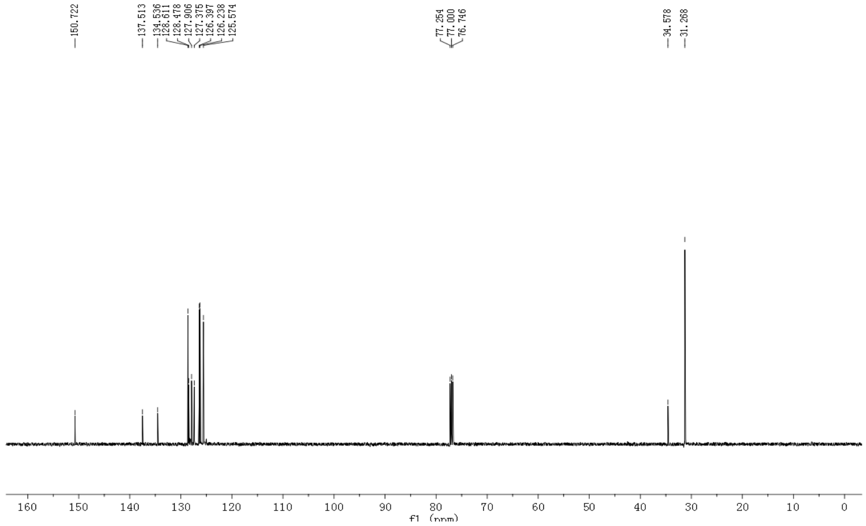

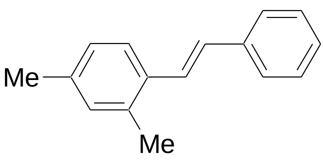

Compound **4l** [8]: a colorless liquid; ^{1}H NMR (500 MHz, $CDCl_3$) δ 2.31 (s, 3H), 2.39 (s, 3H), 6.95 (d, *J* = 16.0 Hz, 1H), 6.99–7.02 (m, 2H), 7.22–7.36 (m, 4H), 7.48–7.51 (m, 3H); ^{13}C NMR (125 MHz, $CDCl_3$) δ 19.8, 21.1, 125.3, 126.4, 126.9, 127.4, 128.6, 129.0, 131.2, 133.5, 135.6, 137.3, 137.8.

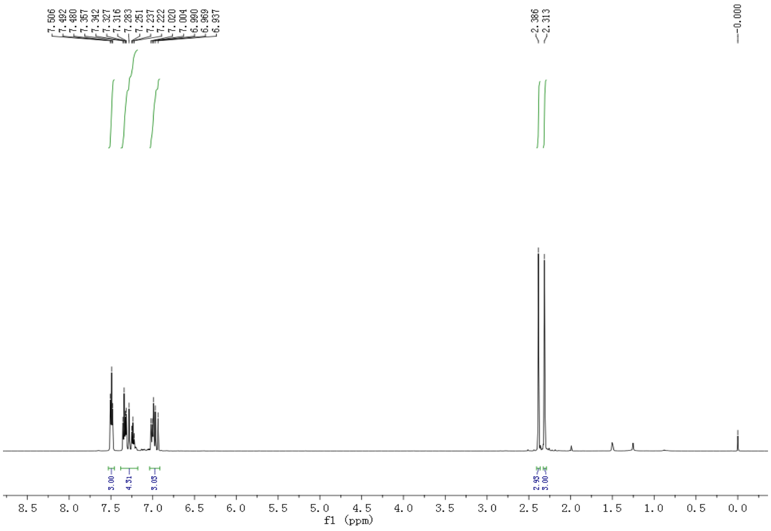

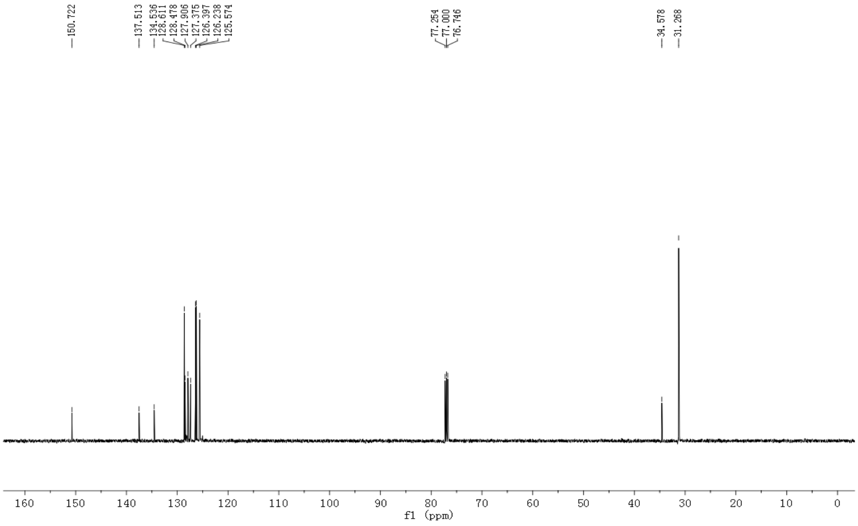

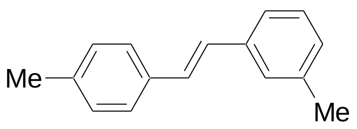

Compound **4m** [3]: a white solid; ^{1}H NMR (500 MHz, $CDCl_3$) δ 2.35 (s, 3H), 2.37 (s, 3H), 7.03 (d, *J* = 15.9 Hz, 1H), 7.06–7.09 (m, 2H), 7.16 (d, *J* = 7.5 Hz, 2H), 7.22–7.32 (m, 3H), 7.40 (d, *J* = 8.0 Hz, 2H); ^{13}C NMR (125 MHz, $CDCl_3$) δ 21.2, 21.4, 123.6, 126.4, 127.1, 127.8, 128.2, 128.4, 128.5, 129.4, 134.6, 137.4, 137.5, 138.2.

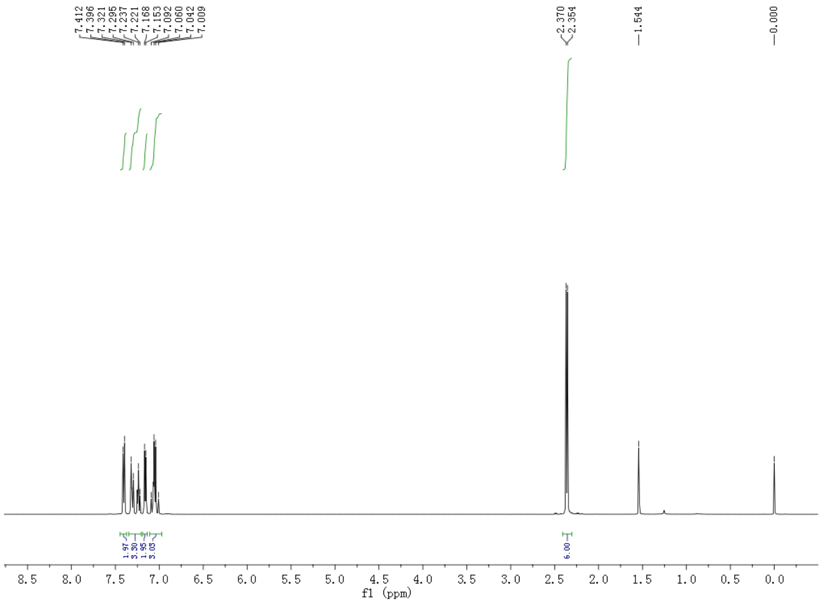

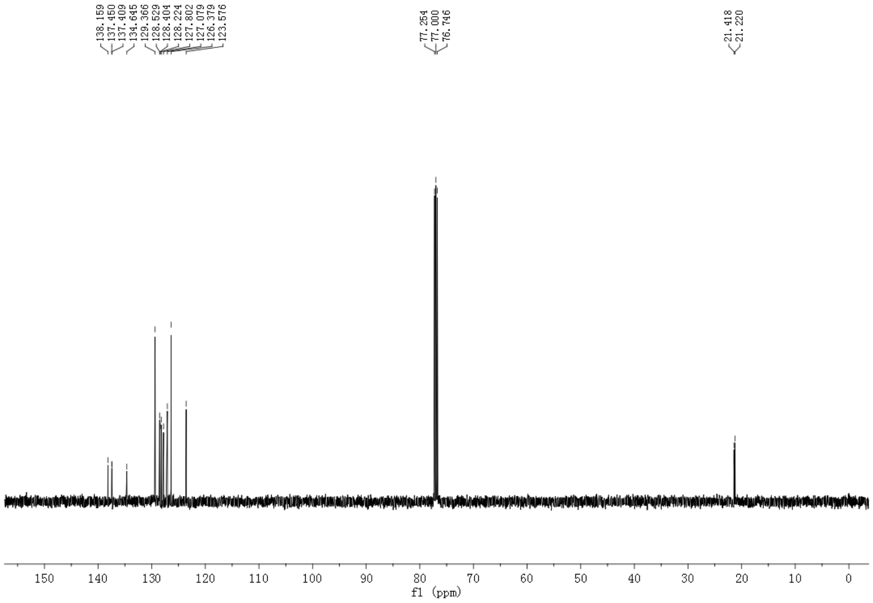

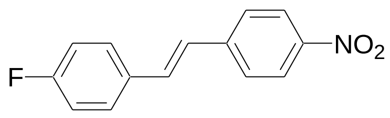

Compound **4n** [9]: a yellow solid; ^{1}H NMR (500 MHz, $CDCl_3$) δ 7.06 (d, J = 16.5 Hz, 1H), 7.10 (d, J = 7.0 Hz, 2H), 7.22 (d, J = 16.5 Hz, 1H), 7.52 (t, J = 7.0 Hz, 2H), 7.61 (d, J = 8.0 Hz, 2H), 8.22 (d, J = 8.0 Hz, 2H); ^{13}C NMR (125 MHz, $CDCl_3$) δ 115.9 (d, $J_{C\text{-}F}$ = 21.8 Hz), 124.1, 126.1 (d, $J_{C\text{-}F}$ = 2.3 Hz), 126.8, 128.6 (d, $J_{C\text{-}F}$ = 8.1 Hz), 132.0, 132.4, 143.7, 146.8, 163.0 (d, $J_{C\text{-}F}$ = 247.9 Hz).

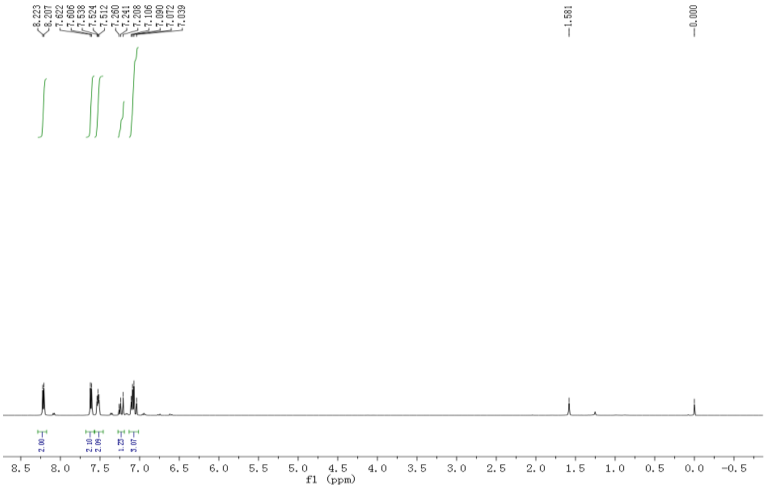

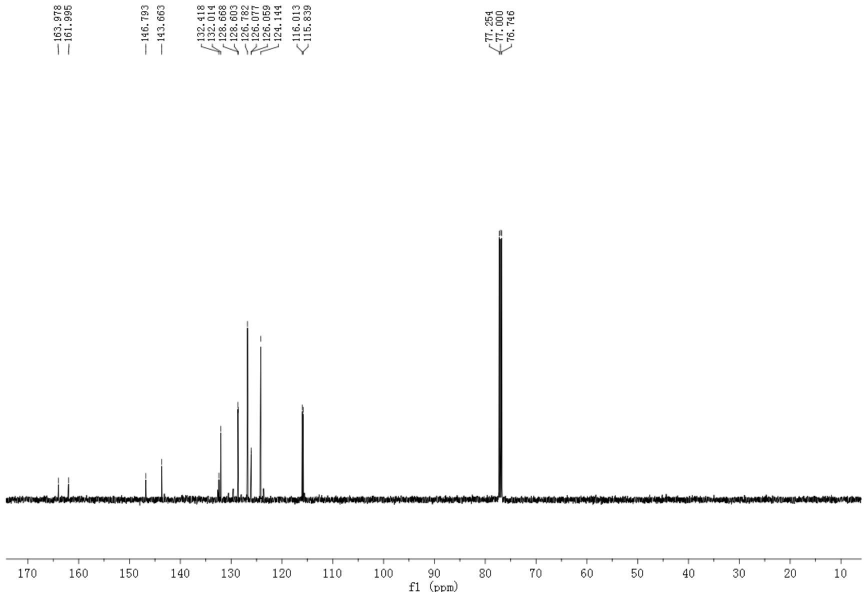

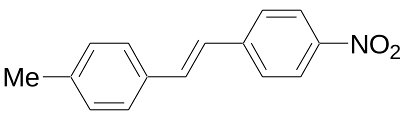

Compound **4o** [3]: a yellow solid; ^{1}H NMR (500 MHz, $CDCl_3$) δ 2.38 (s, 3H), 7.09 (d, *J* = 16.0 Hz, 1H), 7.22 (d, *J* = 7.5 Hz, 2H), 7.24 (d, *J* = 16.0 Hz, 1H), 7.44 (d, *J* = 7.5 Hz, 2H), 7.61 (d, *J* = 8.5 Hz, 2H), 8.20 (d, *J* = 8.5 Hz, 2H); ^{13}C NMR (125 MHz, $CDCl_3$) δ 21.3, 124.1, 125.3, 126.7, 127.0, 129.6, 133.3, 133.4, 139.0, 144.1, 146.6.

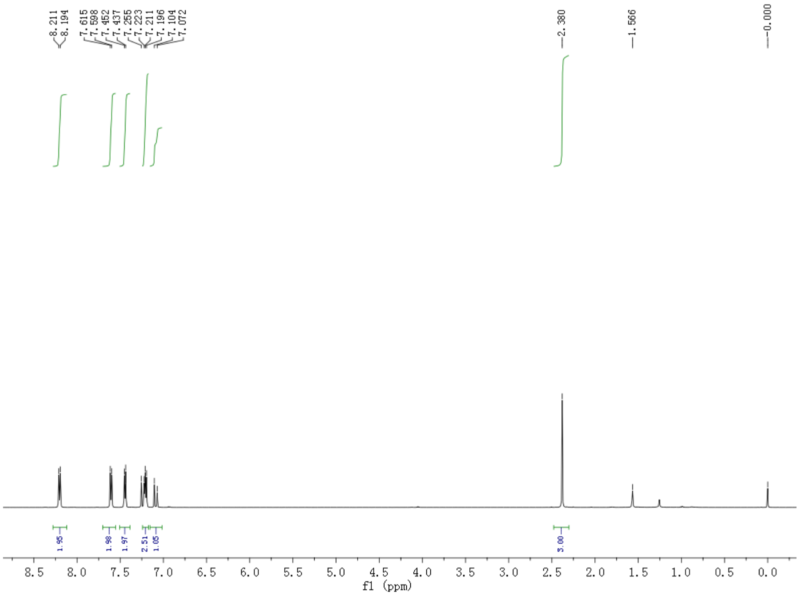

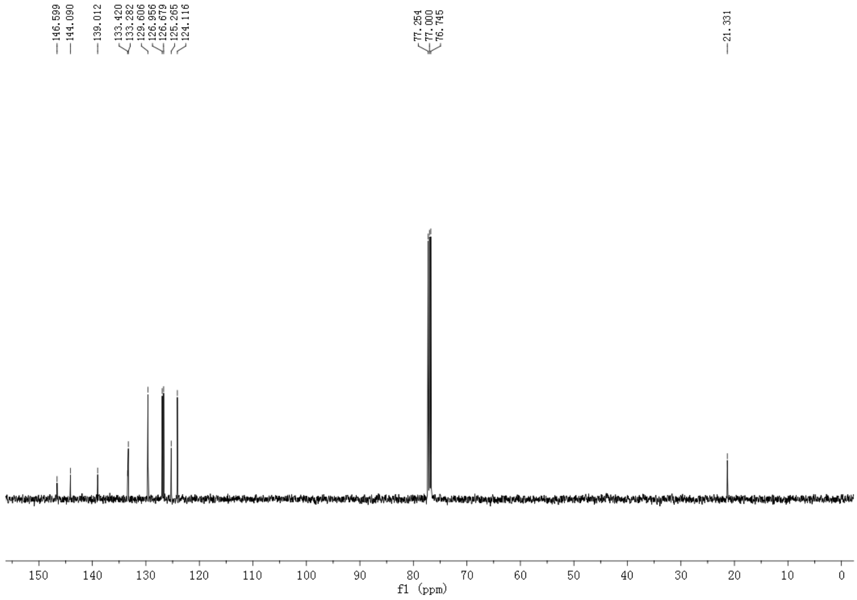

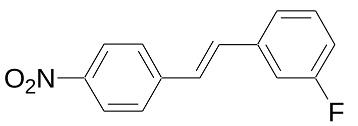

Compound **4p** [10]: a yellow solid; ^{1}H NMR (300 MHz, $CDCl_3$) δ 7.02 (t, J = 8.0 Hz, 1H), 7.13 (d, J = 16.0 Hz, 1H), 7.22 (d, J = 16.0 Hz, 1H), 7.31–7.38 (m, 3H), 7.63 (d, J = 8.5 Hz, 2H), 8.22 (d, J = 8.5 Hz, 2H); ^{13}C NMR (125 MHz, $CDCl_3$) δ 113.2 (d, $J_{C\text{-}F}$ = 21.9 Hz), 115.6 (d, $J_{C\text{-}F}$ = 21.3 Hz), 123.0, 124.1, 127.0, 127.6, 130.4 (d, $J_{C\text{-}F}$ = 8.3 Hz), 131.9, 138.5 (d, $J_{C\text{-}F}$ = 7.5 Hz), 143.3, 147.0, 163.2 (d, $J_{C\text{-}F}$ = 244.6 Hz).

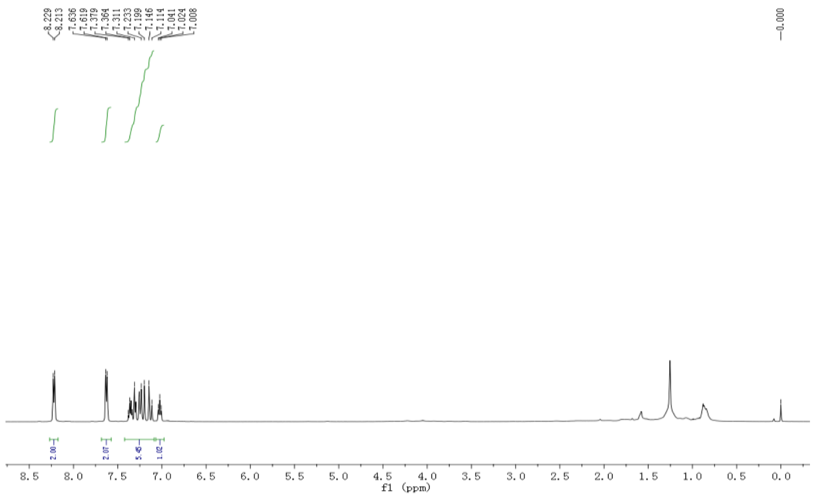

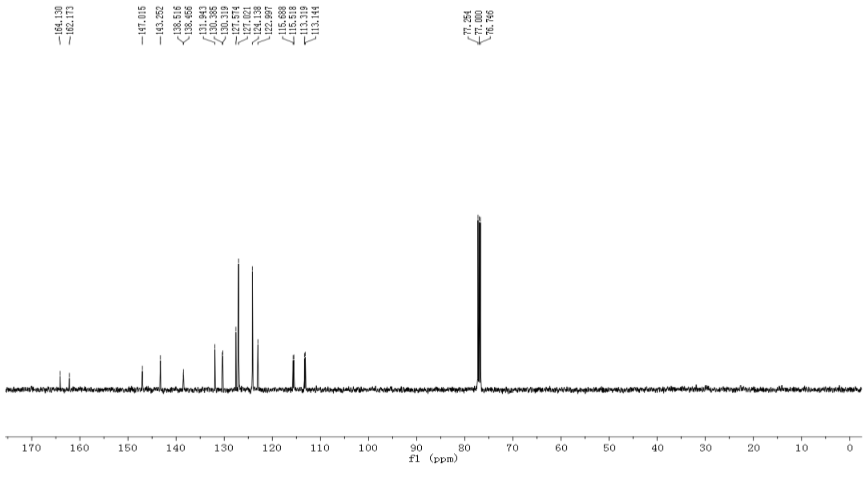

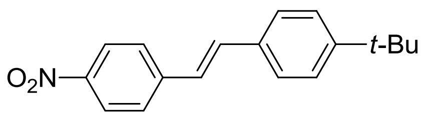

Compound **4q** [11]: a yellow solid; ^{1}H NMR (300 MHz, $CDCl_3$) δ 1.34 (s, 9H), 7.10 (d, *J* = 16.0 Hz, 1H), 7.25 (d, *J* = 16.0 Hz, 1H), 7.42 (d, *J* = 8.0 Hz, 2H), 7.49 (d, *J* = 8.0 Hz, 2H), 7.61 (d, *J* = 8.0 Hz, 2H), 8.20 (d, *J* = 8.0 Hz, 2H); ^{13}C NMR (125 MHz, $CDCl_3$) δ 31.2, 34.8, 124.1, 125.5, 125.8, 126.7, 126.8, 133.2, 133.4, 144.1, 146.6, 152.2.

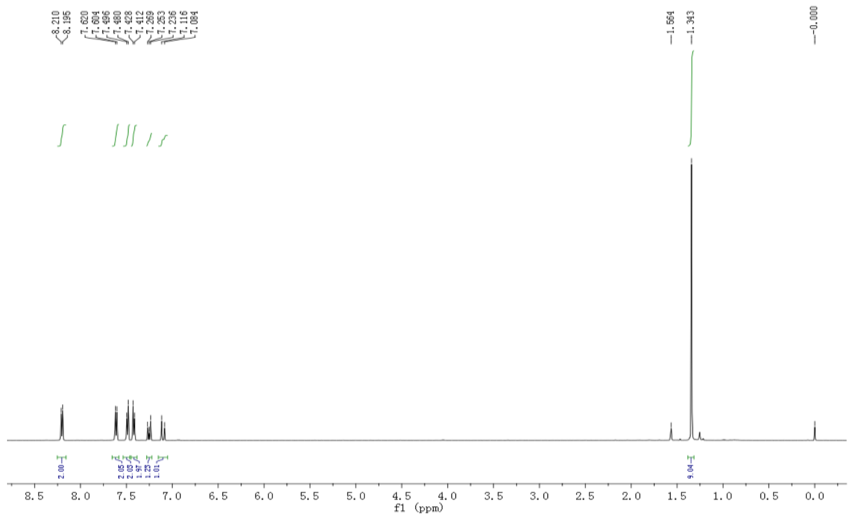

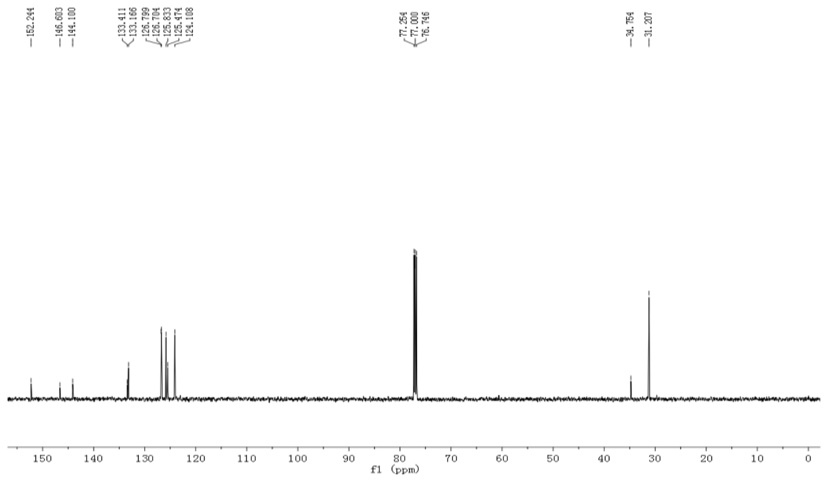

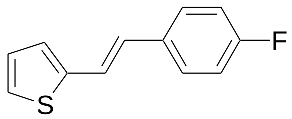

Compound **4r** [12]: a yellow solid; ^{1}H NMR (300 MHz, $CDCl_3$) δ 6.88 (d, *J* = 16.2 Hz, 1H), 6.98–7.11 (m, 5H), 7.18 (dd, *J* = 4.2, 5.1 Hz, 1H), 7.38–7.45 (m, 2H). ^{13}C NMR (125 MHz, $CDCl_3$) δ 115.6 (d, $J_{C\text{-}F}$ = 21.6 Hz), 124.3, 126.1, 127.1, 127.6, 127.8 (d, $J_{C\text{-}F}$ = 8.0 Hz), 133.1, 142.7, 162.3 (d, $J_{C\text{-}F}$ = 245.9 Hz).

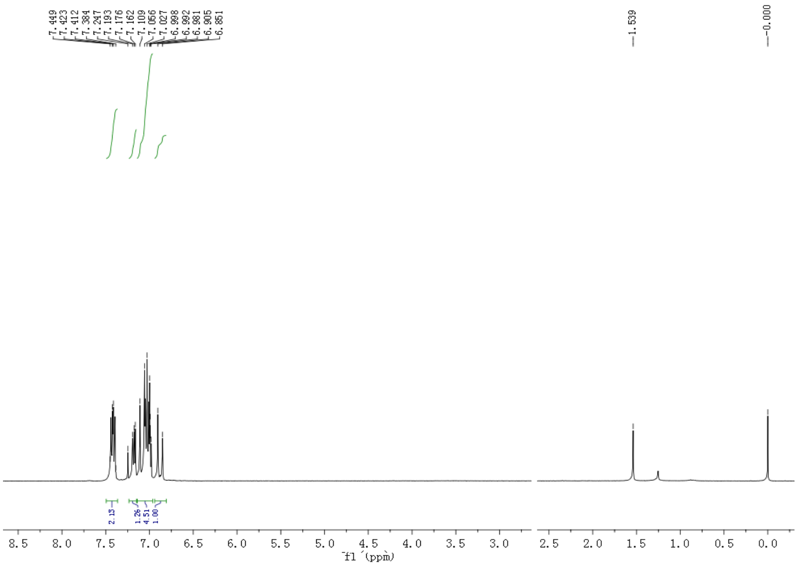

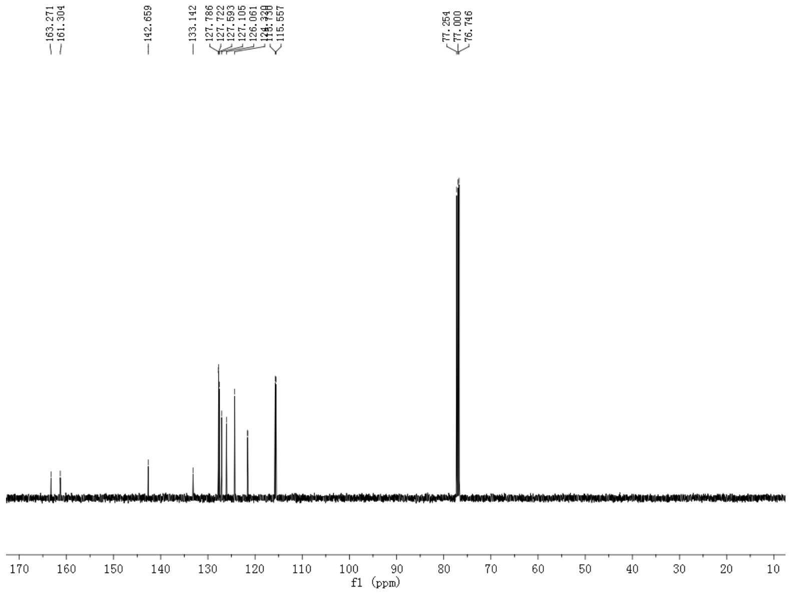

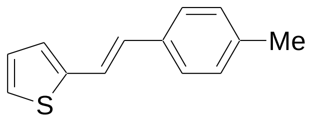

Compound **4s** [12]: a yellow solid; ^{1}H NMR (500 MHz, $CDCl_3$) δ 2.35 (s, 3H), 6.90 (d, J = 16.0 Hz, 1H), 6.99 (t, J = 3.5 Hz, 1H), 7.04 (d, J = 3.5 Hz, 1H), 7.14–7.24 (m, 4H), 7.36–7.49 (d, J = 8.0 Hz, 2H); ^{13}C NMR (125 MHz, $CDCl_3$) δ 21.2, 120.8, 124.0, 125.7, 126.2, 127.5, 128.3, 129.4, 134.2, 137.5, 143.1.

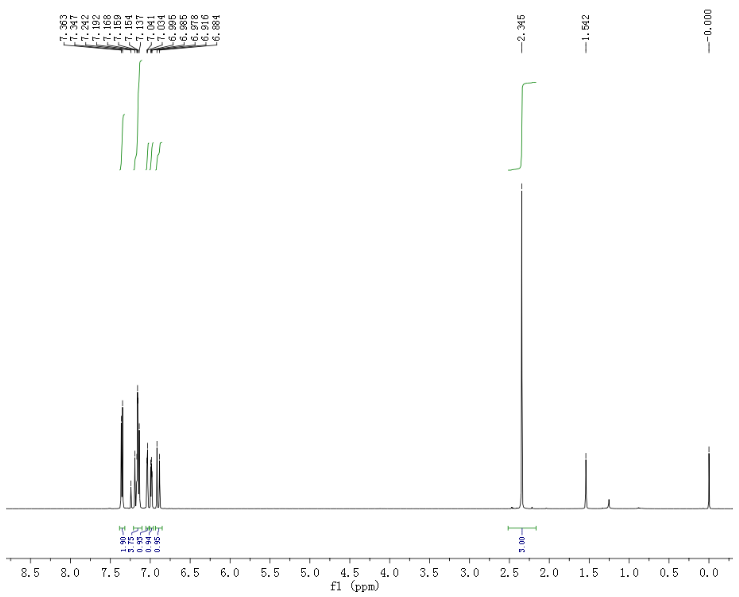

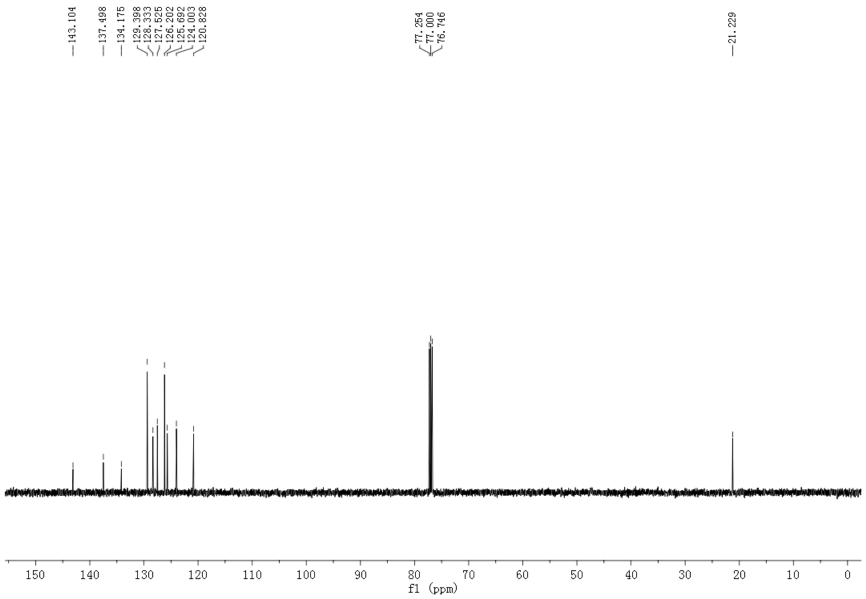

References

1. Xu, H.-J.; Zhao, Y.-Q.; Zhou, X.-F. *J. Org. Chem.* **2011**, *76*, 8036-8041.

2. Leng, Y.-T.; Yang, F.; Wei, K.; Wu, Y.-J. *Tetrahedron* **2010**, *66*, 1244-1248.

3. Inomata, S.; Hiroki, H.; Terashima, T.; Ogata, K.; Fukuzawa, S.-i. *Tetrahedron* **2011**, *67*, 7263-7267.

4. Wang, W.; Yang, Q.; Zhou, R.; Fu, H.-Y.; Li, R.-X.; Li, X.-J. *J. Organomet. Chem.* **2012**, *697*, 1-5.

5. Blakemore, J. D.; Chalkley, M. J.; Farnaby, J. H.; Guard, L. M.; Hazari, N.; Incarvito, C. D.; Luzik, E. D., Jr.; Suh, H. W. *Organometallics* **2011**, *30*, 1818-1829.

6. Mochida, S.; Hirano, K.; Satoh, T.; Miura, M. *J. Org. Chem.* **2011**, *76*, 3024-3033.

7. Wu, K.-M.; Huang, C.-A.; Peng, K.-F.; Chen, C.-T. *Tetrahedron* **2005**, *61*, 9679-9687.

8. Kabalka, G. W.; Al-Masum, M.; Mereddy, A. R.; Dadush, E. *Tetrahedron Lett.* **2006**, *47*, 1133-1136.

9. Wu, S.; Ma, H.-C.; Jia, X.-J.; Zhong, Y.-M.; Lei, Z.-Q. *Tetrahedron* **2011**, *67*, 250-256.

10. Simons, L. J.; Caprathe, B. W.; Callahan, M.; Graham, J. M.; Kimura, T.; Lai, Y.-J.; LeVine, H., III; Lipinski, W.; Sakkab, A.; Tasaki, Y.; Walker, L. C.; Yasunaga, T.; Ye, Y.-Y.; Zhuang, N.; Augelli-Szafran, C. E. *Bioorg. Med. Chem. Lett.* **2009**, *19*, 654-657.

11. Sawoo, S.; Srimani, D.; Dutta, P.; Lahiri, R.; Sarkar, A. *Tetrahedron* **2009**, *65*, 4367-4374.

12. Ho, J.-H.; Ho, T.-I. *J. Chin. Chem. Soc.* **2003**, *50*, 109-114.
